# Psychometric Properties of the Polish Version of Task and Ego Orientation in Sport Questionnaire (TEOSQ)

**DOI:** 10.3390/ijerph17103593

**Published:** 2020-05-20

**Authors:** Maciej Tomczak, Małgorzata Walczak, Paweł Kleka, Aleksandra Walczak, Łukasz Bojkowski

**Affiliations:** 1 Department of Psychology, Poznan University of Physical Education, 61-871 Poznan, Poland; walczak@awf.poznan.pl (M.W.); bojkowski@awf.poznan.pl (Ł.B.); 2 Faculty of Psychology and Cognitive Sciences, Adam Mickiewicz University in Poznan, 60-568 Poznan, Poland; pawel.kleka@amu.edu.pl; 3 Faculty of Medicine, Poznan University of Medical Sciences, 61-701 Poznan, Poland; aleksandra.i.walczak@gmail.com

**Keywords:** psychometric properties, reliability, validity, TEOSQ

## Abstract

The main aim of the study was to assess the psychometric properties of the Polish version of the task and ego orientation in sport questionnaire (TEOSQ). The study covered 651 athletes aged 19.2 years, SD (Standard deviation) = 2.21. The task and ego orientation in sport questionnaire (TEOSQ) and sport motivation scale (SMS-28) were used. Cronbach’s Alpha for the ego subscale was 0.84, and for the task subscale 0.81 (McDonald’s omega was 0.84, 0.82 respectively). The reliability of the test-retest with two weeks interval was ICC (Intraclass correlation coefficient) = 0.86 for ego and ICC = 0.86 for task. Initially, the two-factor model was not fully fitted (CFI (Comparative fit index) = 0.84), however the model with correlated errors for selected test items was well fitted to data (CFI = 0.95). Statistically significant, positive correlations between the task orientation and the intrinsic motivation components were obtained. Additionally, individual athletes had higher scores on the ego factor and lower scores on the task factor than the team athletes. These effects were moderated by the level of participation and occurred among high-performance athletes. Due to satisfactory reliability and validity indicators the Polish version of the task and ego orientation in sport questionnaire (TEOSQ) can be used both for scientific research and in the individual diagnostics of athletes.

## 1. Introduction

Among the various theories of motivation Nichols’ [1,2] achievement goal theory holds an important place in the field of the sport psychology. It assumes that a person’s perception of success and his or her competence depends on the two main orientations, i.e., task and ego. Task-oriented athletes perceive success through opportunities to develop their competences. They are oriented towards skill development, which is the primary source of their motivation to maintain activity. They are characterized by intrinsic motivation, in which the pleasure of performing tasks is of great motivational importance. They are capable of making a long-term effort because the perspective of developing their competences gives them the source of the pleasure. They tend to focus on skill development regardless of the performance level of other athletes. They do not experience the need to compare their performance with other people’s. In turn, ego-oriented people define success by capability to perform better than other athletes. They assess their competence by comparing their level with that of other athletes. Being better than others is their primary source of motivation to act. Therefore, they tend to compare their level of performance with others. Their drive towards competence development may significantly decrease when they achieve a higher level of performance than other athletes [1,2,3,4,5,6].

In reference to the two orientations, the task and ego orientation in sport questionnaire (TEOSQ) was created [7,8]. Currently, research on ego and task orientation in the field of sport activities is generally carried out using the task and ego orientation in sport questionnaire (TEOSQ) and perceptions of success in sport questionnaire (POSQ) [9]. They are among the most cited motivational methods in sport [10]. Adaptations of the TEOSQ both in the area of sport and/or physical education have been implemented in various countries, e.g., Spain, Italy, Greece, Portugal, Japan, Croatia, the UK, Korea. In the conducted studies, indicators concerning the reliability and validity of the tool were obtained. At first, two assumed factors (task and ego) were found in exploratory factor analysis [7]. Subsequent studies carried out in different countries in the area of sporting activity generally showed a satisfactory fit of the two-factor model to the data [4,5,11,12]. However, the basic two-factor model was not always fully fitted. For example, in one of the initial studies, TEOSQ models for different groups were improved by allowing error correlation for some test items [3]. Kim and Gill [13] also improved the TOESQ model estimated on the sample of young Korean athletes by removing a few questions. The model estimated on the Mexican sample also did not achieve a good fit. It has been improved by allowing correlation for some errors of items [14].

Lochbaum et al. [9] reviewed studies on commonly assumed hypotheses regarding the relationship of ego and task (TEOSQ, POSQ questionnaires) with other variables. Taking into account research by TEOSQ questionnaire, they pointed out that women had a little advantage in task orientation than men and men did not have an advantage in ego as was assumed. Moreover, elite athletes did not have significantly higher scores on the task orientation subscale than non-elite athletes. They also did not have significantly lower scores on the ego orientation subscale than non-elite athletes. On the other hand, individual athletes presented higher scores on the ego factor than team athletes. Athletes from countries with individualistic culture had a higher result on the task orientation subscale than those from countries with collectivistic culture. Moreover, they did not have a lower result on the ego orientation subscale than those from countries with collectivistic culture [9]. As achievement goal theory concerns the motivational sphere, the relationships between task, ego, and other motivational variables have also been systematically presented. For example, relatively strong positive relationships between task orientation and intrinsic motivation components from the sport motivation scale were obtained [15,16,17,18,19], while ego orientation was positively related to external motives [15,19]. It is also clearly noted that task-oriented people believe that sports performance depends on effort, while ego-oriented people believe that sport effects depend on ability [20]. In addition, it has been shown that task orientation is positively associated with other variables, e.g., mastery [7,21] or cooperation [7,22]. Task orientation and ego orientation were also associated indirectly with well-being by athletic social connectedness (mediator), with task being positively and ego being negatively associated with social connectedness [23].

As a result of the need for research in goal orientation among athletes in Poland, the main aim of the study was to assess the psychometric properties of the Polish version of task and ego orientation in sport questionnaire (TEOSQ).

## 2. Method

### 2.1. Participants

The study covered 651 athletes (348 high-performance athletes, 303 recreational athletes, 247 women, 404 men, 348 individual athletes and 303 team athletes) aged 19.2 years (SD = 2.21).

### 2.2. Measurement Tools

The task and ego orientations in sport questionnaire (TEOSQ) was used [8]. The scale consists of 13 questions (6 concerns ego orientation and 7 task orientation items). The role of the examined person is to indicate to what extent the statement on a scale of 1–5 refers to him/her. The original version of TEOSQ was translated into Polish by a bilingual expert. Then this version was translated back by another bilingual expert. The original and translated English versions of test were compared by a committee of six bilingual experts, i.e., sports psychologists, trainers, PE teachers. Then the expert’s comments on cultural differences were taken into account (minor changes were made to the final Polish version). These studies do not require the opinion of the bioethics committee in Poland because they do not have the characteristics of a medical experiment (they contain only questionnaires). The psychometric indicators of the Polish version of the scale were presented at the 13th FEPSAC Congress [24].

The sport motivation scale (SMS-28) by Pelletier et al. [25] in the Polish adaptation by Walczak and Tomczak [26] was also used. In order to assess the TEOSQ validity, correlations between the TEOSQ subscales and the sport motivation scale were determined. This scale contains 28 questions. They relate to the following components: to know (intrinsic motivation), to accomplish (intrinsic motivation), to experience stimulation (intrinsic motivation), identification (extrinsic motivation), introjection (extrinsic motivation), external regulation (extrinsic motivation), amotivation. Relatively high correlations between the TEOSQ task orientation and the intrinsic motivation components of the SMS-28 questionnaire were predicted.

### 2.3. Statistical Analysis

In order to determine the reliability of the scale, Cronbach’s alpha and McDonald’s omega indexes were calculated. Alpha is sensitive to the multidimensionality of the construct therefore omega index was calculated additionally. This is particularly important for the overall result, which is often not one-dimensional. However, it is recommended to present two coefficients at the same time, which also allows to compare these two values. Acceptable values for both alpha and omega are from 0.70 to 0.95 [27,28].

To assess the quality of individual test items, item-rest correlation was used and the information function from multidimensional graded response model of item response theory (IRT) was presented. Generally, IRT is used to determine the relationship between latent traits and its measurable indicators that can be, e.g., answers for questionnaire questions. Values of information function and information curves are presented. The curves show the information values for a given level of trait. Curves indicating that the low value of information for a given answer category show low precision in a specific area. There are no thresholds set for results and information function values. Information curves allow to assess the precision of an item in the context of other values. A higher value of information function is adequate to smaller standard error and higher reliability of item [29,30,31].

Then, in order to assess the factor structure of the Polish version, confirmatory factor analysis was conducted. Confirmatory factor analysis is used to check how hypothetically assumed factor structure (model) fits to empirical data. Comparative fit index (CFI) and Tucker–Lewis index (TLI) values 0.95 and above as well as root mean square error approximation (RMSEA) and standardized root mean square residual (SRMR) values below 0.06 and 0.08 respectively, indicate a good model fit [32,33]. Less restrictive criteria are taken into account sometimes, e.g., CFI and TLI values above 0.90 and RMSEA values below 0.08 are considered as enough (or minimal) for acceptance of the model [34,35]. The analysis was performed using R environment and AMOS software (version 26) (IBM, Armonk, NY, USA). The first estimated model was consistent with theoretical framework which assumed existence of two orthogonal factors (ego, task). Then, as a result of inadequate fitting of the basic model with data, modification indexes were analysed. Next, the model with the released paths for errors of some items was assessed.

In order to assess the relationship between the task and ego orientation of the TEOSQ questionnaire and the sport motivation scale components, r-Pearson correlations were used (this analysis was conducted on 596 people). Then, the canonical analysis was used to assess the total relationship between the variables, where ego subscale and task subscale were used as outcome variables, and SMS-28 scale components were used as independent variables. This analysis allows to analyse two sets of variables determining the total relationship between independent and dependent variables (as a generalization of multiple regression). As a result of the analysis, so-called canonical variables are created as combinations of input dependent and independent variables. The relationship between canonical variables is determined based on so-called canonical correlation. Canonical weights and canonical factor loadings correspond to the relationships of dependent and independent variables with canonical variables. Canonical weights are interpreted similarly to regression coefficients and canonical factor loadings are interpreted as simple correlations [36]. Analysis was performed using Statistica software (13.3 version) (TIBCO Statistica™, Palo Alto, CA, USA).

In order to assess the TEOSQ, ego and task were also compared by gender, sport type (individual, team), and level of participation (high performance, recreational). A three-way analysis of variance was used to compare women and men, high-performance and recreational athletes as well as individual and team athletes in terms of goal orientation. In the case of significant effects, the post-hoc analysis was performed with the Bonferroni correction. In order to assess the reliability of the test-retest based on the examination of the same people in a two-week interval, intraclass correlation coefficient (ICC) correlations were used (*n* = 51 athletes).

## 3. Results

### 3.1. Analysis of Internal Consistency and the Item-Rest Correlations

**Figure 1:**
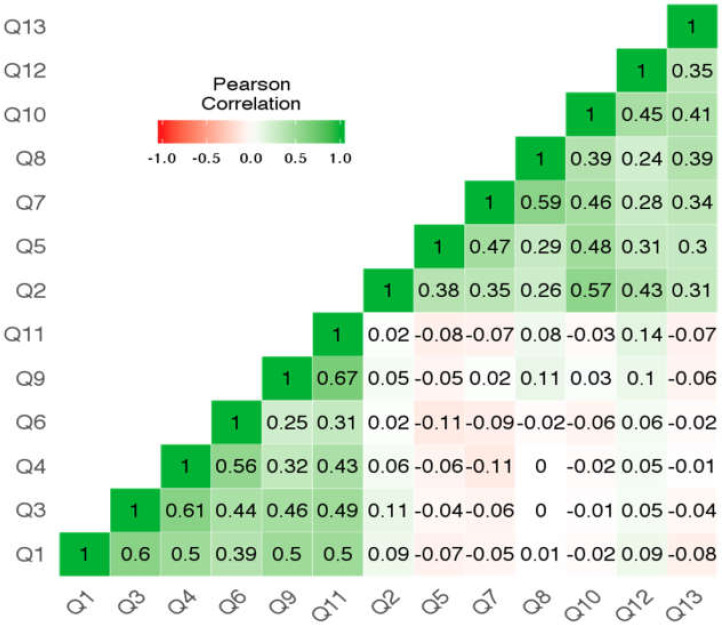
Heatmap of item-item correlations.

For the ego subscale the Cronbach’s alpha is 0.84 and the correlations item-rest are respectively: Q1—0.66, Q3—0.70, Q4—0.64, Q6—0.50, Q9—0.59, Q11—0.64. For the Task subscale the Cronbach’s alpha is 0.81 and the correlation item-rest are respectively: Q2—0.54, Q5—0.53, Q7—0.60, Q8—0.51, Q10—0.68, Q12—0.49, Q13—0.50. Reliability of the whole test and ego and task subscales based on McDonald’s omega was 0.77, 0.84, 0.82 respectively. Items are more highly correlated within their own subscales: for ego-green colour, lower left corner of Figure 1, for task-upper right corner of Figure 1). Correlations with items from another subscale are low–lower right corner of Figure 1.

### 3.2. Reliability Analysis by Test-Retest

The reliability of the test-retest method with a two-week interval (*n* = 51) was ICC_3_ = 0.86, *p* < 0.001 for ego and ICC_3_ = 0.86, *p* < 0.001 for task subscale.

### 3.3. Analysis of the Item Reliability Based on IRT Model

**Figure 2:**
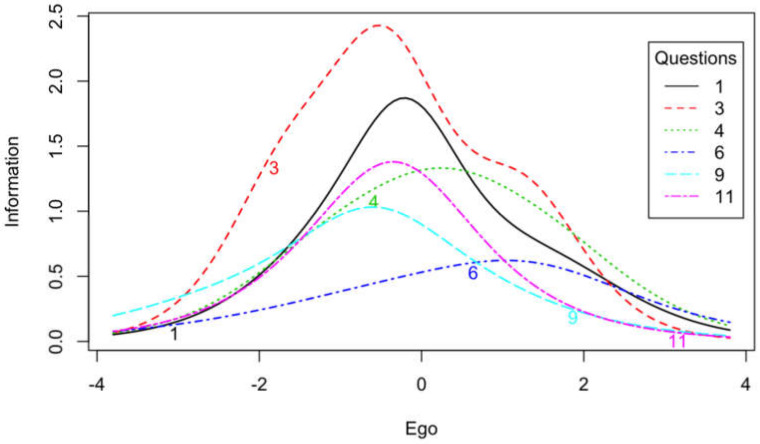
Item information curves for ego items.

**Figure 3:**
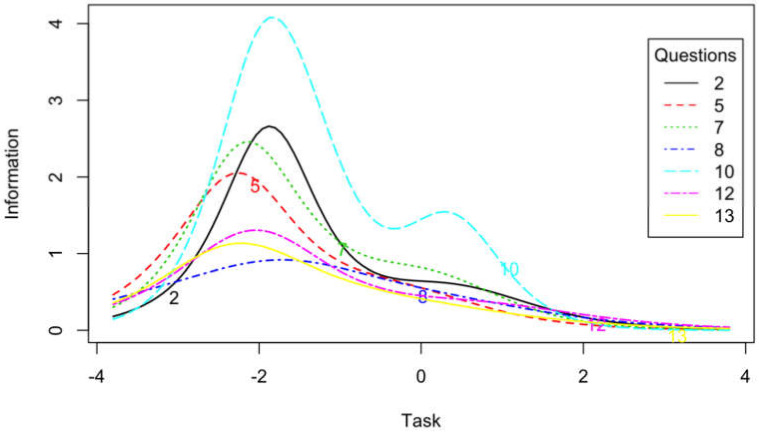
Item information curves for task items.

We also evaluated the reliability of particular items based on the results obtained in the items response theory (IRT) analysis using generalized partial credit model (GPCM) scaling. The obtained results of precision values of particular positions estimated on the basis of the results of the informative function are presented in Table 1 and Figure 2 and Figure 3. In the ego subscale, items generally provided more information in middle values of trait than the high and low values of traits. The least information provided item 6 (Figure 2). On the other hand, in the case of the task subscale, items contained more information for a low-level trait than a high-level trait. The less information than other items provided items 8, 12, and 13 (Figure 3).

### 3.4. Analysis of the Factor Structure of TEOSQ

**Figure 4:**
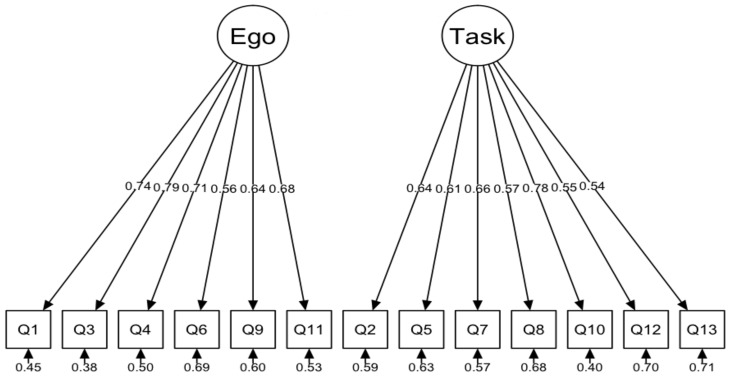
Factor model of TEOSQ questionnaire (Chi-sq = 539.67, df = 65, *p* < 0.001, CFI = 0.840, TLI = 0.808, SRMR = 0.068, RMSEA = 0.106, CI.90 [0.098–0.114]).

**Figure 5:**
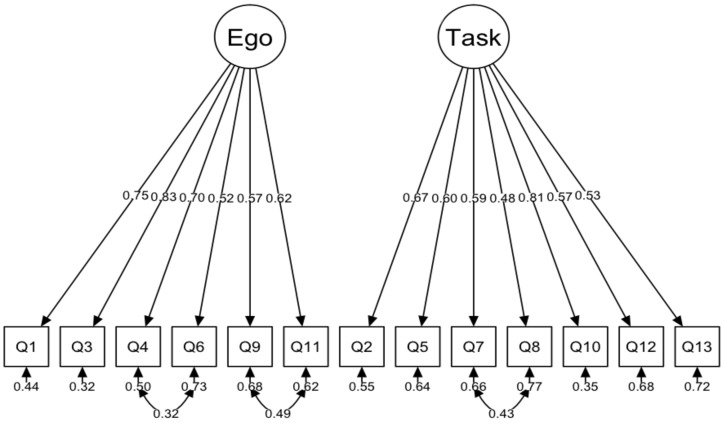
TEOSQ Questionnaire factor model with covariance for errors: 4–6, 7–8, 9-11, (Chi-sq = 217.43, df = 62, *p* < 0.001, CFI = 0.948, TLI = 0.934, SRMR = 0.052, RMSEA = 0.062, CI.90 [0.053–0.071]).

The basic, two-factor model did not get a good fit for the data (Figure 4). Analysis of the modification indexes indicates that the three indexes for the covariance of errors of items have very high values, i.e., the index for the covariance of errors of items 9 and 11 (140.93), 7 and 8 (96.90), and 4 and 6 (63.92) (there are more high values, however, considering the highest three values for errors leads to a satisfactory fit). In the next analysis, the covariance between errors was allowed. The corrected model is characterized by a relatively good fit to the data (Figure 5).

### 3.5. Relationships between TEOSQ Subscales and SMS-28 Components

There were statistically significant positive, relatively strong correlations between the subscale task and the intrinsic motivation components (intrinsic motivation to know, intrinsic motivation to accomplish, intrinsic motivation to experience stimulation) as well as positive, but weaker correlations with the external motivation components (identification, introjection, external regulation). A negative correlation between task and amotivation was also observed. There was a statistically significant, positive, weak correlation between ego and external regulation (Table 2). Then, a canonical analysis was performed.

Both canonical correlations are statistically significant. The sets of variables for the canonical variables are presented in Table 3 and Table 4. The first pair of canonical variables is mainly made up of subscale task and intrinsic motivation components. The second pair of variables is mainly made up of the ego and the external regulation.

### 3.6. The Relationship between Sport Type, Level of Participation, Gender and TEOSQ Subscales

**Figure 6:**
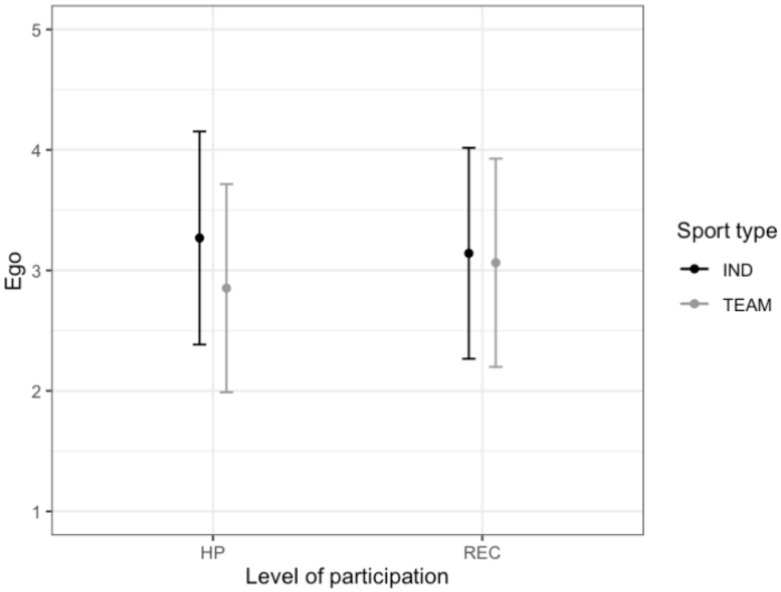
The relationship between the level of participation (High performance—HP, Recreational—REC), type of sport (Individual—IND, Team—TEAM) and ego orientation (mean and standard deviation).

A statistically significant main effect of the sport type was noted for ego subscale (F (1.643) = 14.02, *p* < 0.001, η_p_^2^ = 0.021). Individual athletes (IND) (M = 3.22, SD = 0.88) had higher scores on the ego factor than the team athletes (TEAM) (M = 2.98, SD = 0.87). There was also a statistically significant interaction effect of sport type and level of participation (F (1.643) = 7.51, *p* < 0.01, η_p_^2^ = 0.012) (Figure 6). Among high performance, individual athletes (M = 3.27, SD = 0.88) had higher scores on the ego factor than the team athletes (M = 2.85, SD = 0.86) (*p* < 0.001). There were no statistically significant differences among recreational athletes (IND: M = 3.14, SD = 0.88; TEAM: M = 3.06, SD = 0.86; *p* > 0.05) (Figure 6). The main effects of the level of participation (F (1.643) = 0.61, *p* > 0.05, Gender (F (1.643) = 0.16, *p* > 0.05, and interaction effects sport type*gender (F (1.643) = 3.74, *p* > 0.05), level of participation*gender (F (1.643)1.83, *p* > 0.05) and sport type*level of participation*gender (F (1.643) = 0.67, *p* > 0.05) were not significant.

**Figure 7:**
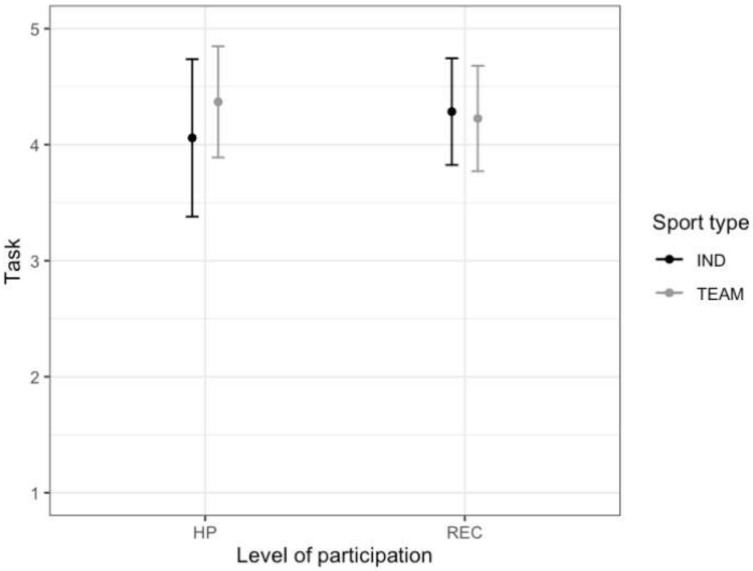
The relationship between the level of participation (High performance—HP, Recreational—REC), type of sport (Individual—IND, Team—TEAM) and task orientation (mean and standard deviation).

In the case of the task subscale, the main effect of the sport type was obtained (F (1.643) = 7.58, *p* < 0.01, η_p_^2^ = 0.012). Team athletes (M = 4.28, SD = 0.47) had higher scores on the task factor than individual athletes (M = 4.14, SD = 0.62). The effect of interaction between level of participation and sport type was also noted (F (1.643) = 14.82, p < 0.001, η_p_^2^ = 0.023) (Figure 7). Among high-performance athletes, team athletes had higher scores on the task factor than individual athletes (TEAM: M = 4.37, SD = 0.48, IND: M = 4.06, SD = 0.68; p < 0.001). There were no significant differences in task orientation among recreational athletes (TEAM: M = 4.23, SD = 0.45, IND: M = 4.28, SD = 0.46; *p* > 0.05). Moreover, among individual athletes, recreational athletes presented higher level of task orientation than high-performance athletes (*p* < 0.01) (Figure 7). However, no statistically significant main effects were observed for gender: F (1.643) = 0.91, *p* > 0.05, level of participation: F (1.643) = 0.64, *p* > 0.05, and interaction effects: sport type * gender: F (1.643) = 0.03, *p* > 0.05, level of participation * gender: F (1.643) = 0.60, *p* > 0.05, sport type * level of participation* gender: F (1.643) = 0.57, *p* > 0.05.

## 4. Discussion

In the case of the Polish version of the TEOSQ scale, relatively high (above 0.80) internal consistency indicators were obtained for both the task and ego subscales. They were relatively similar to the values obtained by other researchers [3,4,12]. High values of the McDonald’s Omega index indicate the high reliability of the scale. Item-rest correlations indicate the high quality of test items. As IRT results indicate, items from the task subscale provide more information about low-level traits. In turn, items from the ego subscale contain more information about the middle values of the characteristic. In addition, items no. 6 (ego subscale), 8, 13, and 12 (task subscale) provide less information than other items for different levels of traits. However, due to the rarity of IRT analysis usage, it is difficult to compare the TEOSQ IRT analysis results to those of other authors. These issues require further research to verify the obtained results. The high reliability of the Polish version of the tool was also demonstrated by the test-retest method. High correlation coefficients for both subscales were obtained in the study carried out on the same group of athletes at a two-week interval.

The results of confirmatory factor analysis did not indicate a good fit of the model to data which, according to theoretical framework, assumed two orthogonal factors. The analysis of modification indexes indicated that it was worth releasing correlations between some errors, i.e., in the task scale, items 7 (I learn a new skill by trying hard) and 8 (I work really hard). In the ego subscale, it was item 4 (The others cannot do as well as me), item 6 (Others mess up, and I do not), item 9 (I score the most points/goals/hits), and item 11 (I am the best). This model fitted well to the data. These questions are quite similar in content, and they are relatively close together. For these reasons, the respondents may answer them similarly. However, it is worth considering that the model with error correlations for some items is based on analysis of modification indexes performed after the main analysis. These indexes are analysed in an exploratory rather than theoretical way (the decision is made only by high index values). Therefore, the model obtained may be specific to a given sample of data collected, e.g., in a specific cultural context, which limits the possibility of generalization. Correlations between errors for some TEOSQ scale questions are not unusual. In the case of Chi and Duda’s study [3], the model did not initially fully fit to the data. Correlations between some questions were therefore allowed (items 9 and 11 and 2 and 10). 

To validate TEOSQ, correlations between task, ego, and SMS-28 component were calculated. Relatively high positive relationships between task orientation and intrinsic motivation components were observed. These results were also supported by canonical analysis which concern total relationship between variables. These are in line with the predictions and previously obtained results [16,17,18]. So, we can conclude that task and intrinsic motivation measure a relatively similar empirical construct.

The analysis of intergroup comparisons showed that individual athletes have stronger ego orientation than the team athletes. Similar results have already been obtained by other researchers, as shown meta-analysis of the TEOSQ study conducted in different countries [9]. Such a result is consistent with the predictions. However, the interaction analysis showed that this effect mainly relates to high-performance athletes. Team athletes, on the other hand, have higher scores on the task factor than individual athletes. This effect applies particularly to high-performance athletes. Stronger ego orientation and weaker task orientation observed in particular among individual high-performance athletes may be related to the specific situation in professional sports. In professional sports, performance and competition are more important than in recreational sports. The low efficiency of an athlete in professional sports carries real consequences for him/her. Adaptation of competitors to such a situation may be significantly related to the type of orientation specific to a particular type of sport (individual/team). It may manifest itself, e.g., through higher scores on the ego factor for individual athletes and higher scores on the task factor for team athletes.

## 5. Conclusions

Good reliability indicators and satisfactory validity for the Polish version of the task and ego orientation in sport questionnaire (TEOSQ) were obtained. The questionnaire can be used in the Polish cultural context both for scientific research and for individual diagnostics.

## Figures and Tables

**Table 1:** Informative function for TEOSQ items.

TEOSQ Items	IRT Information of Ability in Range (−4,4)	
	Raw	Percent
Q1	5.59	0.99
Q3	8.08	1.00
Q4	5.40	0.98
Q6	2.81	0.92
Q9	3.72	0.94
Q11	4.08	0.98
Q2	5.74	0.98
Q5	5.22	0.95
Q7	6.22	0.98
Q8	3.70	0.89
Q10	9.49	1.00
Q12	4.13	0.94
Q13	3.59	0.93

**Table 2:** Correlations of ego subscale and task subscale with Sport Motivation Scale (SMS-28) components.

Variables	Subscales of Sport Motivation Scale						
	KN	AC	ES	IDF	INT	ER	AMT
EGO	−0.03	−0.03	−0.02	0.05	0.01	0.22	0.05
	*p* = 0.523	*p* = 0.506	*p* = 0.625	*p* = 0.180	*p* = 0.735	*p* < 0.001	*p* = 0.187
TASK	0.54	0.59	0.52	0.26	0.36	0.11	−0.30
	*p* < 0.001	*p* < 0.001	*p* < 0.001	*p* < 0.001	*p* < 0.001	*p* = 0.009	*p* < 0.001

*n* = 596, KN—intrinsic motivation to know; AC—intrinsic motivation to accomplish; ES—intrinsic motivation to experience stimulation; IDF—identification; INT—introjection; ER—external regulation; AMT—amotivation.

**Table 3:** Loadings for canonical variables.

Variables	Canonical Variables	
	1	2
EGO	−0.11	−0.99
TASK	1.00	−0.09
Canonical Correlation	0.62 (*p* < 0.001)	0.24 (*p* < 0.001)
Variables	1	2
KN	0.86	−0.14
AC	0.95	−0.16
ES	0.84	−0.16
IDF	0.40	−0.35
INT	0.56	−0.22
ER	0.14	−0.95
AMT	−0.49	−0.08

KN—intrinsic motivation to know; AC—intrinsic motivation to accomplish; ES—intrinsic motivation to experience stimulation; IDF—identification; INT—introjection; ER—external regulation; AMT—amotivation.

**Table 4:** Weighs for canonical variables.

Variables	Canonical Variables	
	1	2
EGO	−0.09	−1.00
TASK	0.99	−0.11
Canonical Correlation	0.62 (*p* < 0.001)	0.24 (*p* < 0.001)
Variables	1	2
KN	0.33	0.17
AC	0.53	−0.12
ES	0.18	−0.02
IDF	−0.06	0.21
INT	0.09	0.20
ER	−0.16	−1.19
AMT	−0.12	0.10

KN—intrinsic motivation to know; AC—intrinsic motivation to accomplish; ES—intrinsic motivation to experience stimulation; IDF—identification; INT—introjection; ER—external regulation; AMT—amotivation.
